# Production of microalgal-based carbon encapsulated iron nanoparticles (ME-nFe) to remove heavy metals in wastewater

**DOI:** 10.1007/s11356-022-22506-x

**Published:** 2022-08-25

**Authors:** Marco Mantovani, Elena Collina, Marina Lasagni, Francesca Marazzi, Valeria Mezzanotte

**Affiliations:** Department of Earth and Environmental Sciences (DISAT), Università degli Studi di Milano—Bicocca, P.zza della Scienza 1, 20126 Milano, Italy

**Keywords:** Microalgae, Hydrothermal carbonization, Iron nanoparticles, Metal, Remediation

## Abstract

The integration of microalgae-bacteria consortia within existing wastewater treatment plants as alternative biological treatment could be an interesting option to improve the sustainability of these facilities. However, the fate of the produced biomass is decisive to make that option economically attractive. The present study aimed to valorize the microalgae grown at a pilot scale and used for the treatment of the centrate from municipal sewage sludge, producing microalgal-based iron nanoparticles (ME-nFe), by hydrothermal carbonization. The final product had high carbon content, strong sorbent power, and reducing properties, due to the presence of zerovalent iron. Different synthesis conditions were tested, comparing iron (III) nitrate nonahydrate (Fe (NO_3_)_3_·9H_2_O) and ammonium iron (III) sulfate dodecahydrate (NH_4_ Fe (SO_4_)_2_·12 H_2_O) as iron sources, four different Fe/C molar ratios (0.02, 0.05, 0.1, 0.2), and three process temperatures (180, 200, 225 °C). Based on the characterization of all the prototypes, the best one (having a specific area of 110 m^2^g^−1^) was chosen and tested for the removal of selected heavy metals by Jar tests. The removal of copper, zinc, cadmium, and nickel from the treated effluent from the wastewater treatment plant was 99.6%, 97.8%, 96.4%, and 80.3%, respectively, also for very low starting concentrations (1 mg L^*−*1^). The removal of total chromium, on the contrary, was only 12.4%. Thanks to the magnetic properties, the same batch of ME-nFe was recovered and used effectively for three consecutive Jar tests.

## Introduction

The need for advanced technologies to remove specific pollutants from wastewater is pushing research all over the world. The problem is very complex due to the extremely wide variety of compounds of concern, their interactions, and, especially, their very low concentrations. Oxidative processes are most commonly applied, and they involve the use of various chemicals as oxidants (ozone, hydrogen peroxide, etc.) or as catalysts (iron) and/or of physical reactions based on UV irradiation or ultrasounds. Advanced oxidation processes (AOP) typically exploit ozone and/or hydroxyl radicals which must be generated in situ (Deng and Zhao 2015). In some cases, chemical oxidation does not completely mineralize organic pollutants but transforms them into more biodegradable compounds (such as alcohols and carboxylic acids) which can be subsequently removed by biological processes (Esplugas et al. 2007). Another possibility is the removal of micropollutants by adsorption, which partly occurs also in conventional activated sludge process, using activated carbon or other kinds of adsorbents as, for instance, coconut-shell activated carbon (CCAC), porous β-cyclodextrin polymer (CDP), and CDP coated on cellulose microcrystal (CDP@CMC) adsorbents (Ling et al. 2020) or insoluble polymers of β-cyclodextrin (Alsbaiee et al. 2016).

Heavy metals are effectively removed by conventional activated sludge processes, but their residual concentrations in the effluents are often too high with respect to the acceptable levels in the receiving water bodies (Magni et al. 2015). As is the case for most pollutants, the removal is particularly hard when the initial concentrations are low (Saeed et al. 2005; Lim and Aris 2014). Tertiary coagulation-flocculation has variable results according to the specific metal and to the selected coagulant (Hargreaves et al. 2018), while adsorption seems to be effective and is the main pathway for metal removal also in biological activated sludge processes, in constructed wetlands, and microalgae-based processes (Fu and Wang 2011). The variability of removal performances related, among other things, also to the single metal properties and to the initial concentration, is anyway a common feature. Good results can be obtained by membrane filtration, but the cost of such processes is still too high for a large-scale application in municipal contexts.

All the above-mentioned processes present strong limitations due to high capital and operational costs and performances that are often not so reliable. Thus, further research on this topic is needed.

A series of evidence exists about the possibility of exploiting zero-valent iron in advanced oxidation processes (AOP), due to its great efficiency as an electron donor to activate free radicals (Li et al. 2019; Ambika et al. 2020) or as a reductive agent to remove pollutants (Arvaniti et al. 2015). Zero-valent iron (ZVI) is non-toxic, abundant, cheap, and easy to produce. It is a reactive metal and an effective reducing agent and can be used for removing both organic and inorganic pollutants (e.g., chlorinated organics, pharmaceuticals, metals, textile dyestuffs) (Crane and Scott 2014; Hoch et al. 2008; Sunkara et al. 2010; Qiu et al. 2018). This is an important point related to the above-mentioned problem of the broad variety of emerging compounds and metal pollution, whose main common aspects are the low concentrations, the long persistence, and the high environmental hazard but whose chemical and physico-chemical properties are extremely different.

The application of zero-valent iron nanoparticles (nZVI) in wastewater treatment is very interesting due to the high reactivity, reducing properties, and high specific surface area. However, a full-scale approach is limited due to its lack of stability, easy aggregation, and difficulty in separating nZVI from the treated solution. Also, the interaction between nZVI and oxygen causes too fast aging of the nanoparticles, limiting the long-term effectiveness (Calderon and Fullana 2015). So, research has also considered coated or encapsulated ZVI nanoparticles. Just to cite some cases, a significant removal (38–96%) of perfluorinated compounds (PFC) was obtained by Arvaniti et al. (2015) using Mg-aminoclay coated nanoscale zero-valent iron (starting pH = 3.0). The maximum efficiency was observed for PFOA (perfluorooctanoic acid), followed, in the order, by PFNA (perfluorononanoic acid), PFOS (perfluorooctane sulfonate), and PFDA (perfluorodecanoic acid) and was attributed both to sorption and to degradation.

nZVI encapsulated in microscale carbon spheres (6–8 μm) via an in situ formation through hydrothermal carbonization (HTC) from an organic compound, followed by self-reduction, has excellent chemical reducing capability of nZVI and high sorption capacity, facilitated by the carbon substrate. HTC is a thermal treatment of an aqueous solution or dispersion of a carbon-containing organic material at moderate temperatures and under pressure, which produces a carbon-rich black solid as an insoluble product (Sevilla and Fuertes 2009). The final carbonaceous product presents a core-shell structure with many functional groups on the external surface that can provide a high reactivity (Hu et al. 2010). The incorporation of nZVI into solid particles results in its easy separation from the aqueous system avoiding aggregation of nZVI (Crane and Scott 2014). Encouraging results for metal removal (Zn, Cu, Ni, Cd, Cr) have been obtained at a lab-scale by Bonaiti et al. (2017) using clarified olive mill waste (OMW) as a source of carbon to produce carbon-encapsulated zerovalent iron nanoparticles (CE-nZVI) by HTC.

Here, the results obtained in the production of microalgal-based carbon-encapsulated iron nanoparticles (ME-nFe) by hydrothermal carbonization and in their lab-scale application for metal removal are described.

The novelty of this research consists in using the microalgae grown on wastewater, directly harvested in a functional microalgal pilot plant in use in the wastewater treatment plant (WWTP) of Bresso-Niguarda (Milan, Italy). As described in Mantovani et al. (2020), microalgae can be exploited to treat the municipal centrate (deriving from the centrifugation of digestate) to decrease the nitrogen concentration which would prevent its discharge into the receiving water body. Nitrogen and organic matter can be reduced due to the synergistic action of microalgae and bacteria: the oxygen produced by the photosynthesis of microalgae can be used by nitrifying bacteria for the conversion of NH_4_-N to NO_2_-N and NO_3_-N. Nitrification followed by the denitrification step is the conventional technology used in the WWTP, but it requires a high oxygen demand that can be lowered by the contribution of microalgae. In a continuous process, the microalgal biomass is separated by centrifugation, and the treated centrate is redirected to the waterline of the conventional WWTP, with lowered nitrogen load. Tua et al. (2021) proposed a life cycle assessment on the Bresso WWTP case study comparing the baseline WWTP to a scenario including an algal unit with a specific area of 0.1 m^2^/IE (2.2 ha). The induced electricity savings (due to the reduced need to provide external oxygen which is supplied by microalgae) is a key factor leading to an environmental improvement in 7 out of 15 indicators compared to the baseline WWTP. The residual algal biomass is mixed with the digestate from sewage sludge prior to the dewatering unit of the WWTP. The LCA evaluated the possibility to valorize the mix for agricultural use as fertilizer or burning the microalgae in a co-incineration process with other bio-solids. New options for the valorization of microalgae grown on wastewater are needed to improve further the environmental sustainability and the economic balance of the described technology.

This paper suggests using it as a carbon substrate for the HTC process. Microalgae were hydrothermally treated in the past to obtain hydrochar for different applications such as energy production and agriculture (Heilmann et al. 2010; Liu et al. 2019) but also in the wastewater treatment field (Peng et al. 2014). However, HTC was never used to obtain carbon-encapsulated iron nanoparticles from microalgae. The goal is to assess the possibility to integrate the low-cost and low-impact algal-based process, used in the WWTP to reduce the nitrogen load inflow coming from municipal centrate, with the recovery and valorization of the microalgae. Through HTC, the microalgal biomass is exploited to produce carbon encapsulated iron nanoparticles to be used for the removal of heavy metals from the WWTP effluent as an alternative to conventional tertiary treatments.”

## Material and methods

### Chemicals

During the study, the following chemical reagents, of analytical grade, were used: iron (III) nitrate nonahydrate (Fe (NO_3_)_3_·9H_2_O) and ammonium iron (III) sulfate dodecahydrate (NH_4_ Fe (SO_4_)_2_·12 H_2_O) (Sigma-Aldrich) as iron source for the production of the ME-nFe; ethanol and methanol (Sigma-Aldrich) for the polyphenols extraction and the nanoparticles washing immediately after the synthesis; Folin-Ciocalteu’s phenol reagent 2 M (Sigma-Aldrich) to detect polyphenols in the extracts; and (HO)_3_C_6_H_2_CO_2_H·H_2_O (Fischer Scientific) to prepare the standards for the calibration curve of gallic acid; ZnCl_2_, CuCl_2_, K_2_Cr_2_O_7_, CdCl_2_∙H_2_O, Ni (NO_3_)_2_∙6H_2_O (Fischer Scientific) were dissolved in Milli-Q® water to prepare all the metal solutions for the adsorption tests (except for the ones made with the effluent from the Bresso-Niguarda wastewater treatment plant (WWTP) instead of Milli-Q® water); zinc powder and 37% HCl (Fischer Scientific) for the determination of zero-valent iron and the calibration of the method; 2% HNO_3_ (Sigma-Aldrich) for the acidification of the samples collected during the Jar tests.

### Biomass cultivation, harvesting, and characterization

The microalgae used as feedstock for the HTC process were collected from a pilot-scale high rate algal pond (HRAP) located at the Bresso-Niguarda WWTP in the suburban area of Milan (Italy) (more details in Mantovani et al. 2020). The centrate from sludge dewatering was used to grow a mixed culture mainly made of *Chlorella* spp. and *Scenedesmus* spp. in a continuous mode from May 2018 to November 2019. The HRAP was covered by a polycarbonate greenhouse to allow algal growth even during the cold winter months. In Summer, the lateral walls of the structure were removed to favor light penetration, leaving only the polycarbonate roof in place to protect the microalgae from heavy rain events. The microalgae were harvested directly at Bresso using an ELECREM 1 110 / 230 V centrifugal clarifier. The microalgal pellet was then concentrated and dried at 50 °C, ground to a fine powder, and stored in glass bottles for the characterization and the subsequent syntheses. The elemental analysis of the microalgae was performed on 6 samples collected from March 2019 to November 2019 using a Perkin Elmer CHNS/O analyzer 2400 153 series II. The focus was set on the carbon percentage as the aim of the overall study was to produce iron nanoparticles encapsulated in carbonaceous support and the optimal Fe/C ratio had to be defined. However, also the hydrogen, nitrogen, and phosphorus contents were assessed to obtain more data concerning the possible time variability of the biomass properties. Phosphorus was determined after acid digestion (using 7 mL of H_2_NO_3_ and 3mL of H_2_O_2_) following the Green algae procedure (DG-EN-25) in a microwave digester (ETHOS1600, Milestone). Due to the important role of polyphenols as reducing agents during the iron nanoparticle production, the total phenolic content (TCP) was also determined, according to the Folin-Ciocalteu method (Choochote et al. 2014; Doria et al. 2012). Briefly, 50 mg of biomass was used for the extraction in 5 mL of methanol in a sonicating bath with 45 min of contact time. The suspension was filtered on 0.45-μm Millipore filters, and the pellet was used for a subsequent extraction following the same steps; 0.1 mL of the obtained filtered solution was then put in a 15-mL falcon test tube adding 0.6 mL of distilled water, 0.5 mL of Folin-Ciocalteu reagent, and 1.5 of sodium carbonate (20%). The volume was made up to 10 mL with distilled water. The solution was put in the dark for 30 min at room temperature and then analyzed spectrophotometrically at 750-nm wavelength. The quantification was based on a calibration curve of gallic acid (0–0.7 mg mL^−1^) (Doria et al. 2012).

### ME-nFe production

The ME-nFe were produced with a single-step synthesis through hydrothermal carbonization according to Sun et al. (2012) and Calderon et al. (2018). In the present work, the carbonaceous feedstock used consisted of microalgal biomass, replacing glucose and olive mill wastewater, respectively. Microalgae were dried and resuspended in 100 mL Milli-Q® water and mixed with an iron salt, testing four different Fe/C molar ratios (0.02, 0.05, 0.1, 0.2), three process temperatures (180, 200, 225 °C), and two salts as iron sources (Fe (NO_3_)_3_·9H_2_O and NH_4_ Fe (SO_4_)_2_·12 H_2_O). Three grams of biomass was used for each run. The tested conditions for the HTC process were chosen according to literature findings (Peng et al. 2014; Calderon et al. 2018). Based on the microalgal carbon content on a dry weight basis (% *Cb*), the g of C (*m*_*c*_) and then the mole of C in the processed microalgae were determined, considering its C atomic weight (12 u), as follows:

$${\displaystyle \begin{array}{l}{m}_{\mathrm{C}}=\frac{3\ast \%{C}_b}{100}\\ {}{mol}_C=\frac{m_C}{12}\end{array}}$$

The amount of iron salt needed was then determined according to the desired Fe/C molar ratio (*r*) and the molar mass of the chosen iron salts (*M*):

$${\displaystyle \begin{array}{l}{mol}_{\mathrm{iron}}=r\ast {mol}_C\\ {}{m}_{\mathrm{iron}\ \mathrm{salt}}={mol}_{\mathrm{iron}}\ast {M}_{\mathrm{iron}\ \mathrm{salt}}\end{array}}$$

The mixture of microalgal biomass and iron salt was then moved to a High-Pressure Laboratory Reactor (BR-300, BERGHOF) and heated, setting the desired temperature ramp for a 3-h residence time. The reactor was then left cooling overnight, and the solid product of the HTC process was recovered by vacuum filtration using a 0.2-μm cellulose acetate filter. The solid fraction was subsequently washed with a 50:50 (v:v) water-ethanol solution to remove the tar and other HTC residues and dried at 80 °C for 12 h. Finally, it was ground and stored in a glass vial. HTC has been also performed without using the iron salt to prepare microalgal-based hydrochars at the same temperature conditions used for the ME-nFe.

### ME-nFe characterization

The total iron content of the ME-nFe (%Fe_tot_) was determined gravimetrically after muffle combustion at 900–1000 °C for 1 h. The presence of oxygen during the thermal treatment led to carbon loss and CO_2_ volatilization:

$${C}_{(s)}+{\mathrm{O}}_{2\ (g)}\to {\mathrm{CO}}_{2\ (g)}$$

At the end of the process, after cooling, the sample was weighted, assuming it was mainly composed of iron oxide. The same treatment was performed on a sample of microalgal-based hydrochar, produced without iron, to assess the ash content deriving from the combustion of the biomass. The zero-valent iron determination (% Fe^0^) was performed by measuring the hydrogen gas formed through the reaction of metallic iron with an excess of hydrochloric acid, according to Calderon et al. (2018). A graduated buret was connected by a polyvinyl chloride pipe to a separating funnel and a glass vial containing a known quantity of nanoparticles. The system was filled with water. The change in the water height in the buret was due to the hydrogen formed when 1 mL of HCl was added with a syringe to the nanoparticles. The gas formation was proportional to the zero-valent iron content of the samples which can be obtained through stoichiometric computation according to the following reaction:

$$2{\mathrm{Fe}}_{(s)}+6{\mathrm{H}\mathrm{CI}}_{(l)}\to 2{\mathrm{Fe}\mathrm{Cl}}_{3\ (s)}+2{\mathrm{Fe}\mathrm{CI}}_{(3)\ (s)}+3{\mathrm{H}}_{2\ (s)}$$

The BET surface area and the pore size distribution of the samples were obtained by physical adsorption on the solid surface of nitrogen gas molecules at 77 K, using a Coulter SA 3100 analyzer at Università degli Studi di Milano. The method for measuring the specific surface area (m^2^ g^−1^) of the material is based on the theory developed by Brunauer, Emmet, and Teller (BET), while the pore size distribution was calculated by the Barret-Joyner-Halenda (BJH) method using the desorption branch of the isotherm. An LEO 1430 Scanning Electron Microscope (SEM) was used to evaluate the size and morphology of the ME-nFe. A Jeol JEM 1220 Transmission Electron Microscope (TEM) (120 KV) was used to verify the iron distribution within the carbonaceous matrix; 0.1 mg of samples was dissolved in 1 mL of Milli-Q® water for the observation. The microscope was connected to a CCD camera Gatan multiscan. Energy dispersive X-ray (EDX) analyses were performed on a Hitachi TM 1000 scanning electron microscope on the most promising samples to check their atomic composition.

### Application of ME-nFe nanoparticles for the removal of heavy metals

Based on the characterization of the produced ME-nFe, the two most promising samples (D1 and N1 which are described in Table 5) were used to test their ability to remove heavy metals (Cu, Zn, Cr, Ni, and Cd) from aqueous solutions by Jar tests. The metals were selected as they are commonly present in wastewater and effluents from conventional wastewater treatment plants.

The first tests were run using a solution containing all the metals (10 mg L^−1^ each) in Milli-Q® water, comparing two samples (D1 and N1) at two different doses: 2 g L^−1^ and 3 g L^−1^. A VELP FC 6S Jar tester and 500-mL beakers were used, setting a continuous stirring at 90 rpm. A blank beaker was used to quantify water evaporation which could slightly affect the concentrations, serving also as a control; 5-mL samples were taken from the beakers at various reaction times and investigated by Inductively Coupled Plasma-Optical Emission Spectroscopy (Optima 7000 DV PerkinElmer) after filtration on 0.2-μm cellulose acetate filters and acidification with nitric acid (2% in volume).

The second series of tests were performed with the same nanoparticle samples and procedures but with a starting metal concentration of 1 mg L^−1^, more realistic for treated effluents, to verify if the effectiveness of the treatment was related to the pollutants concentration.

Then, the same procedures were repeated using the effluent from Bresso WWTP as solvent (instead of Milli-Q water) enriched with 10 mg L^−1^ of the selected metals in the first experiments, and with 1 mg L^−1^ in the following ones. The aim was to investigate the performances of ME-nFe in a real matrix where the competition between dissolved solids and the heavy metals for the active sites of the nanoparticles might occur. The effluent was collected before the disinfection step occurring in Bresso at the end of the waterline. The effluent was collected after filtration and before disinfection. Being a tertiary effluent, it had a low solid content (0.05 ± 0.01 g TSS L^−1^). The residual concentrations of total chromium, nickel, copper, and cadmium were 3.5, 30, 1.8, and 0.9 μg L^−1^, respectively.

All the above-described Jar tests were done in triplicate and lasted 54 h: such a long time was adopted to follow the fate of metals, verifying if the nanoparticles would release the adsorbed metals with time. All the tests were coupled with pH and ORP measures. The Milli-Q test solutions had a starting pH of 5.4 which was not adjusted, while the effluent solutions had a starting pH of 7.

The determination of the point of zero charge (PZC) by the pH drift method was carried out on sample N1 (the most performant one) to better understand the different removal mechanisms of heavy metals. A stock solution of 0.01M NaCl was purged with nitrogen gas in a titration vessel to get rid of dissolved CO_2_. Different samples of it were collected, and pH was corrected to a consecutive integer from 2 to 10 using 0.1M HCl and 0.1 NaOH; 150 mg of ME-nFe was added to the pH-adjusted solutions, and each sample was placed in a horizontal shaker for 24 h. The final pH was measured after getting rid of the ME-nFe and plotted against the starting pH. The intersection between the obtained plot and the pH initial=pH final line is defined as PZC, meaning the pH in which the surface of the adsorbent has a neutral charge, as mentioned in Bhattarai et al. (2020). The presence of zero-valent iron and iron oxide in the nanoparticles was important not only for their reactivity but provided for magnetic properties, allowing easy recovery of the ME-nFe after use. Most of the nanoparticles could be separated from the liquid solution with a neodymium magnet, holding them at the bottom of the beaker while siphoning the solution elsewhere. A centrifugation step (5 min at 5000 rpm) was then performed to recover the small residues of ME-nFe.

To understand the potential of reusing the same nanoparticles, Jar tests were repeated in the same way using the recovered nanoparticles The ME-nFe separated from the treated water were dried in the oven (70 °C for 12 h) after every step. By weighting the solid, it was possible to be sure of the recovery rate of the nanoparticles, and the experiment could be repeated with the same concentration of nanoparticles without additional regeneration steps. Further Jar tests were made till the metal removal decreased to 60%.

## Results and discussion

### Feedstock characteristics

The chemical characteristics of the biomass are crucial during the synthesis of the iron nanoparticles by HTC. According to the results shown in Table 1, the overall composition did not show relevant seasonal variations with an average carbon percentage of 41 ± 4%. This was important as the chemical composition of the microalgal biomass may change in time following some shifting in the microalgal community in terms of species, which can easily occur, especially in outdoor cultivations. Similar considerations apply to the total phenolic content. The results reported in Table 2 show an average of 1.3 ± 0.1 mg g^−1^ gallic acid equivalents, suggesting that a similar reducing power should be expected in time. Table 2 also provides the composition of the microalgal community tested for this project. *Chlorella* spp. and *Scenedesmus* spp. were the main taxa observed in the microalgal suspension, and the first one was always dominant. That stability is reflected also in the TCP content. The measured concentrations are certainly not high with respect to data reported by other Authors: Safafar et al. (2015), for instance, report values up to 6 mg g−^1^ for *Chlorella sorokiniana* even if there are great differences with other species. However, the values in Bresso’s biomass are higher than the one reported by Hemalatha et al. (2013) who measured a TCP content of 0.78 ± 0.03 mg g^−1^ in *Chlorella marina* confirming the wide variation range among different taxa.

**Table 1:** Elemental analysis data on dried samples of microalgal biomass

Samples	C tot. (%)	H tot. (%)	N tot. (%)	P tot. (g kg^−1^)
HRAP 21-03	37.3	7.7	8.3	8.2
HRAP 13-05	40.2	8.1	8.9	7.5
HRAP 14-06	46.9	2.3	9.7	3.2
HRAP 02-07	38.5	7.9	8.8	9.2
HRAP 26-09	38.0	7.9	10.2	9.7
HRAP 08-11	42.3	8.2	9.6	8.9
Average	40.6	7.0	9.3	7.8
Dev. st.	3.6	2.3	0.7	2.3

**Table 2:** Total phenolic content of microalgal biomass*.* The results are expressed as *mg g*^*−1*^ d.w. as gallic acid equivalent. (*n*=3)

Sample	Main taxa	TCP (mg g^−1^)	St. dev.
HRAP 21-03	*Chlorella* spp.	1.23	0.10
HRAP 13-05	*Chlorella* spp. *Scenedesmus* spp.	1.37	0.24
HRAP 28-05	*Chlorella* spp. *Scenedesmus* spp.	1.20	0.15
HRAP 14-06	*Chlorella* spp. *Scenedesmus* spp.	1.41	0.19
HRAP 2-07	*Chlorella* spp. *Scenedesmus* spp.	1.11	0.06
HRAP 25-07	*Chlorella* spp.	1.22	0.07
Average		1.26	0.13

The advantage of the HTC is the possibility to exploit the water content of the biomass. However, the low solid concentration of microalgae entails the need to concentrate the microalgal suspension, obtaining a dense sludge-alike solution. Even if the carbon content and the TCP of Bresso microalgae were quite stable, the standardization of the HTC process was performed using the same stock of dried microalgae (HRAP 14-06) to avoid unexpected effects on the synthesis due to differences in the biomass characteristics.

### ME-nFe characteristics

**Fig. 1:**
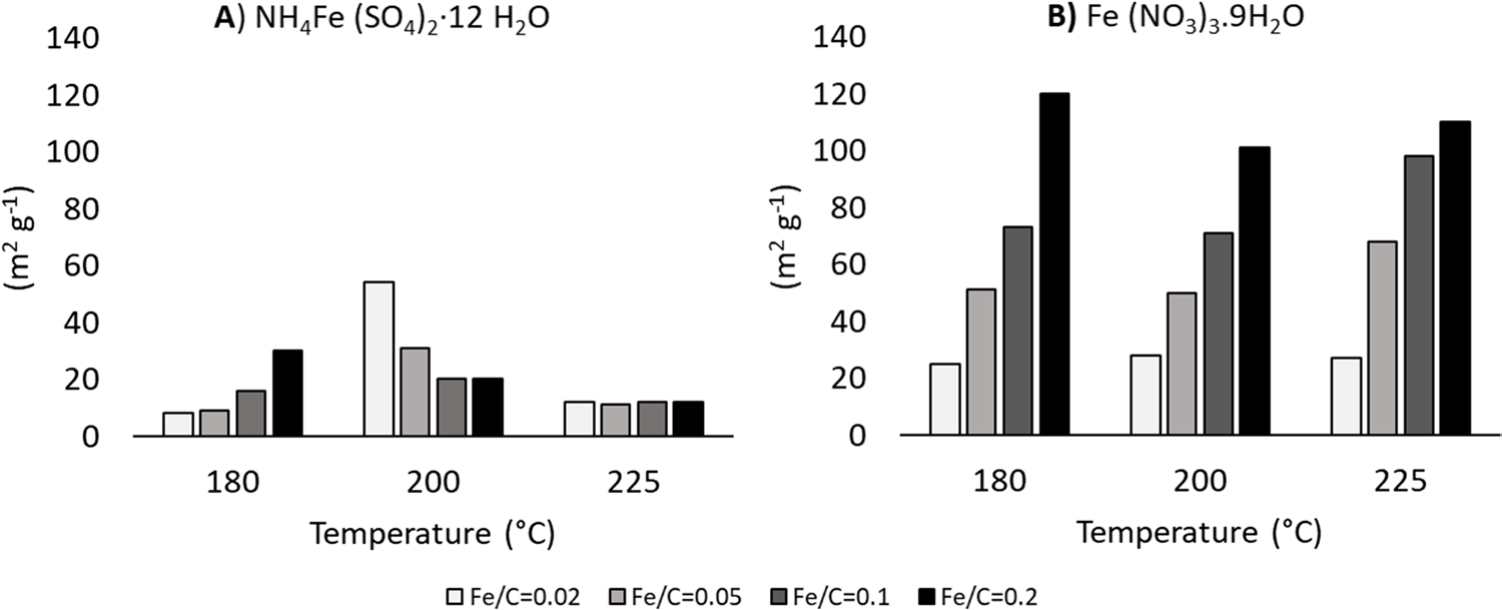
BET surface area vs Fe/C molar ratio for all the produced ME-nFe

The characterization of all the samples was essential to define a final protocol to produce the ME-nFe. Depending on the salt used for the synthesis, different results were obtained. The nanoparticles produced with iron (III) nitrate nonahydrate had BET area up to 120 m^2^g^−1^ that is comparable with literature results on a similar application (Peng et al. 2014), while with ammonium iron (III) sulfate dodecahydrate, the BET area was much lower. Table 3 summarizes the BET surface area of all the produced nanoparticles. As also shown in Fig. 1, at 180 °C the BET surface area increased with increasing Fe/C molar ratio, with both iron salts. For ammonium iron (III) sulfate dodecahydrate, the trend of BET area as a function of Fe/C ratio was opposite at 200 °C, and no trend was observed at 225 °C. The absolute highest value of BET area was obtained with iron (III) nitrate nonahydrate at the lowest temperature (180 °C) with the highest Fe/C ratio. Concerning the samples of microalgal-based hydrochars (without iron), as to be expected, the obtained BET areas were low (18, 22, and 17 m^2^g^−1^ at 180, 200, and 255 °C, respectively). The outcome of the BET analysis suggested ruling out the iron sulfate while microalgal hydrochars (without the addition of the iron) should be better destined for other uses than water remediation. The role of the iron salt was not unexpected as a similar trend was described in the work of Peng et al. (2014) where iron-doped biochar was produced using blue-green microalgae. The reasons for such different results from the types of iron salt are still not clear. Indeed, the specific thermal decomposition of iron nitrate and iron sulfate occurring during the HTC process is different, and this might have important effects on the final texture of the solid product. However, Peng et al. (2014) achieved a satisfying BET area precisely with ammonium iron (III) sulfate dodecahydrate. It is known that the chemical composition of microalgae can sensibly change from species to species but also due to different environmental conditions (Orazova et al. 2014; Batista et al. 2013; Ötleş and Pire 2001), so it is possible to assume that the different results could have depended on the different chemical composition of the microalgae and the interaction between the chemical components of the biomass and the two iron salts during the complex chain-like reactions of HTC.

**Table 3:** List of the ME-nFe samples with the synthesis condition and their BET surface area

Salt	Sample	[Fe/C]	*T* (°C)	BET (m^2^g^−1^)
NH_4_Fe (SO_4_)_2_·12 H_2_O	A	0.02	180	8
NH_4_Fe (SO_4_)_2_·12 H_2_O	B	0.05	180	9
NH_4_Fe (SO_4_)_2_·12 H_2_O	C	0.1	180	16
NH_4_Fe (SO_4_)_2_·12 H_2_O	D	0.2	180	30
NH_4_Fe (SO_4_)_2_·12 H_2_O	E	0.02	200	54
NH_4_Fe (SO_4_)_2_·12 H_2_O	F	0.05	200	31
NH_4_Fe (SO_4_)_2_·12 H_2_O	G	0.1	200	17
NH_4_Fe (SO_4_)_2_·12 H_2_O	H	0.2	200	20
NH_4_Fe (SO_4_)_2_·12 H_2_O	I	0.02	225	12
NH_4_Fe (SO_4_)_2_·12 H_2_O	L	0.05	225	11
NH_4_Fe (SO_4_)_2_·12 H_2_O	M	0.1	225	12
NH_4_Fe (SO_4_)_2_·12 H_2_O	N	0.2	225	12
Fe (NO_3_)_3_^.^9H_2_O	A1	0.02	180	25
Fe (NO_3_)_3_^.^9H_2_O	B1	0.05	180	51
Fe (NO_3_)_3_^.^9H_2_O	C1	0.1	180	73
Fe (NO_3_)_3_^.^9H_2_O	D1	0.2	180	120
Fe (NO_3_)_3_^.^9H_2_O	E1	0.02	200	28
Fe (NO_3_)_3_^.^9H_2_O	F1	0.05	200	50
Fe (NO_3_)_3_^.^9H_2_O	G1	0.1	200	71
Fe (NO_3_)_3_^.^9H_2_O	H1	0.2	200	101
Fe (NO_3_)_3_^.^9H_2_O	I1	0.02	225	27
Fe (NO_3_)_3_^.^9H_2_O	L1	0.05	225	68
Fe (NO_3_)_3_^.^9H_2_O	M1	0.1	225	98
Fe (NO_3_)_3_^.^9H_2_O	N1	0.2	225	110

**Fig. 2:**
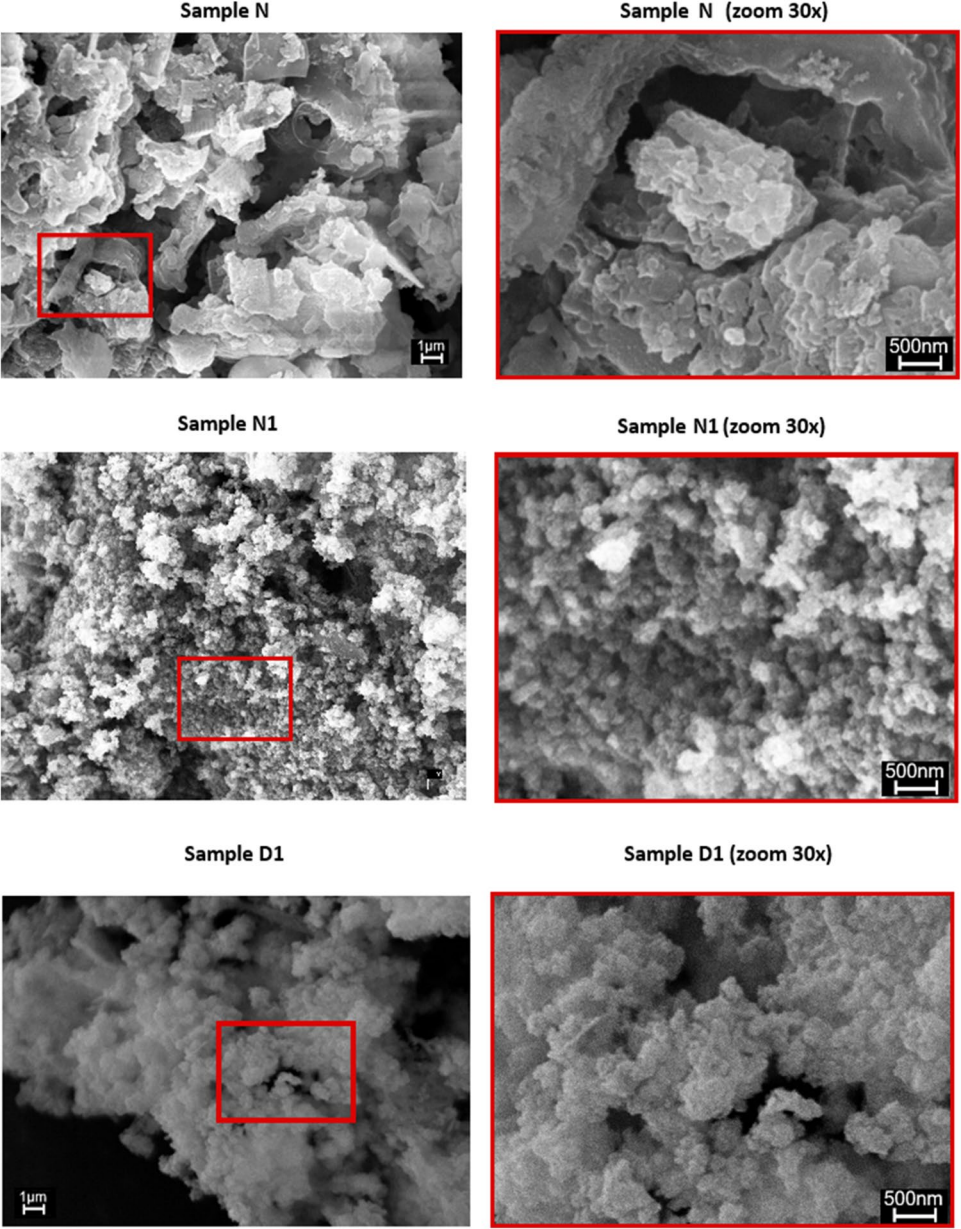
SEM analysis: comparison between the texture of samples N, N1, and D1

The differences detected in the BET surface area were confirmed by SEM analysis showing the morphology of the ME-nFe. The samples made with NH_4_ Fe (SO_4_)_2_·12 H_2_O (Samples A–N) clearly showed a sheet morphology that is consistent with their low BET data, while the nanoparticles produced at the same condition but using Fe(NO_3_)_3_·9H_2_O had a more complex, globular structure (Samples A1–N1). An example of that is given in Fig. 2 where the morphology of samples N, N1, and D1 are compared. In sample N, the texture is more coarse and almost polygonal (this is even more evident in the zoomed picture on the right), while samples N1 and D1 are made of smaller globular aggregates, consistent with the higher BET surface areas.

**Fig. 3:**
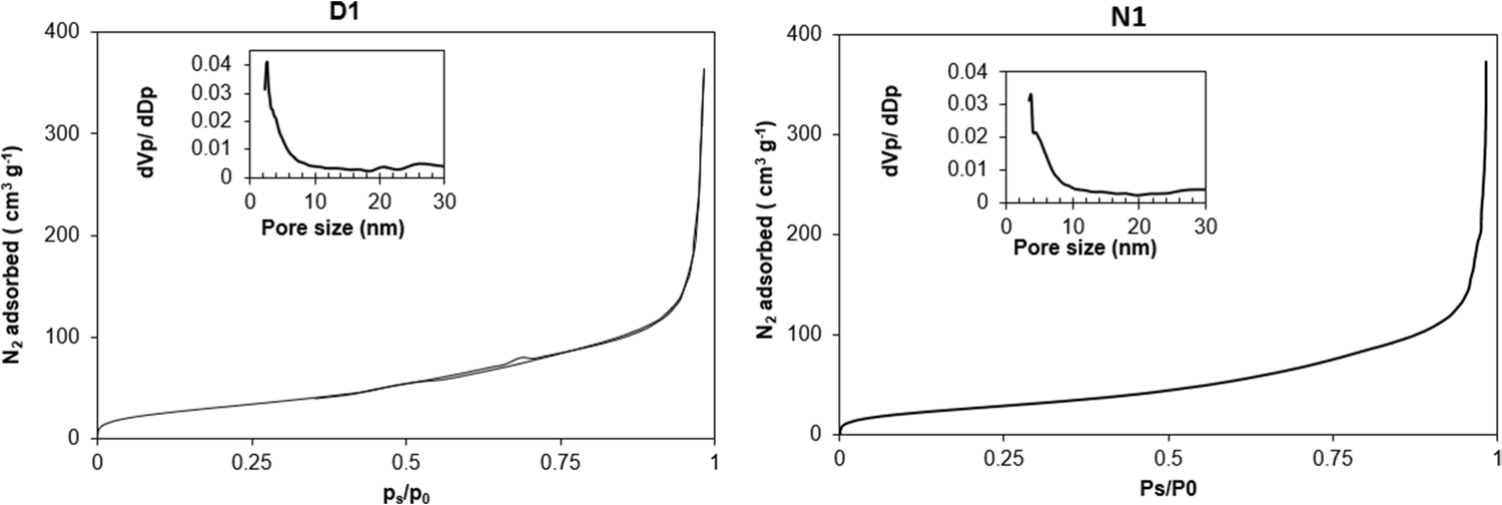
Nitrogen adsorption-desorption isotherms and pore size distribution of the samples D1 and N1

Figure 3 shows the nitrogen adsorption isotherms that were evaluated to calculate the BET area of samples D1 and N1 (the ones having higher BET area), while their pore size distributions were calculated using the Barret-Joyner-Halenda (BJH) method using the desorption branches of the isotherms. Sample D1 and N1 seem similar, almost showing no hysteresis loop. The shape of the isotherms is quite like the type II of IUPAC classification which is typical for nonporous or macroporous materials. A prevalence of pores with diameters higher than 80 nm was detected both in samples D1 and N1 (48 and 45%), but mesoporous were also found with diameters between 6 and 20 nm (9.8 and 10% for D1 and N1, respectively). A considerable number of pores was found to have diameters between 20 and 80 nm (38.4 and 37% for D1 and N1, respectively), while the residual pores were smaller than 6 nm. As for the pore volume, D1 showed a 0.65 cm^3^g^−1^ total pore volume which was slightly higher than the one of N1 (0.58 cm^3^ g^−1^).

Increasing the starting Fe/C molar ratio during the synthesis led to higher total iron incorporation in the final solid products, as suggested in Table 4 which shows the iron concentration in the different samples. The zero-valent iron content (%Fe^0^) ranges between 7 and 14% of the final solid product, comparable to the data obtained by Calderon et al. (2018). Such values were always achieved except for samples A and D. No positive correlation was observed between the starting Fe/C molar ratio and the zero-valent iron content in the nanoparticles. This is not strange as the reduction of the iron salt depends on the reducing properties of the biomass, and, as above explained, the phenolic content of the microalgal biomass was stable in time, providing a similar reducing environment in the reactor for all the synthesis. Accordingly, the iron-reducing efficiency data (% Fe^0^ /Fe _tot_) were higher for lower Fe/C ratios. Higher iron precipitation in the form of iron oxide was achieved by increasing the starting iron dose, while the zero-valent iron concentration remained more stable due to the limiting reducing power of the microalgae.

**Table 4:** Percent of zero-valent iron (% Fe^*0*^), total iron incorporated (% Fe tot), and zero-valent iron incorporation efficiency with respect to the total iron (% Fe^*0*^/Fe tot) in the produced ME-nFe. (*n*=3)

Synthesis		Sample		Fe^0^%	Fe _tot_ %	Fe^0^ /Fe _tot_ %
*T* (°C)	Fe/C	ID	Salt			
180	0.02	A	NH_4_Fe (SO_4_)_2_·12 H_2_O	6.8	28.7	23.7
180	0.05	B		10.6	15.7	67.3
180	0.1	C		8.4	25.9	32.3
180	0.2	D		6.7	40.4	16.5
200	0.02	E		8.8	38.7	22.6
200	0.05	F		14.4	40.2	35.7
200	0.1	G		10.0	58.5	17.5
200	0.2	H		9.5	62.1	15.4
225	0.02	I		9.1	41.5	21.9
225	0.05	L		9.0	42.7	21.1
225	0.1	M		7.4	44.9	16.5
225	0.2	N		8.0	50.4	16.0
180	0.02	A1	Fe (NO3)3 · 9H2O	9.5	17.4	54.4
180	0.05	B1		10.2	26.2	38.8
180	0.1	C1		10.0	35.5	28.1
180	0.2	D1		8.7	42.7	20.4
200	0.02	E1		10.1	15.7	64.8
200	0.05	F1		8.2	23.9	34.1
200	0.1	G1		8.2	34.0	24.1
200	0.2	H1		8.0	41.8	19.2
225	0.02	I1		10.3	44.4	23.2
225	0.05	L1		11.2	45.8	24.5
225	0.1	M1		11.3	64.5	17.6
225	0.2	N1		8.3	66.6	12.5

**Fig. 4:**
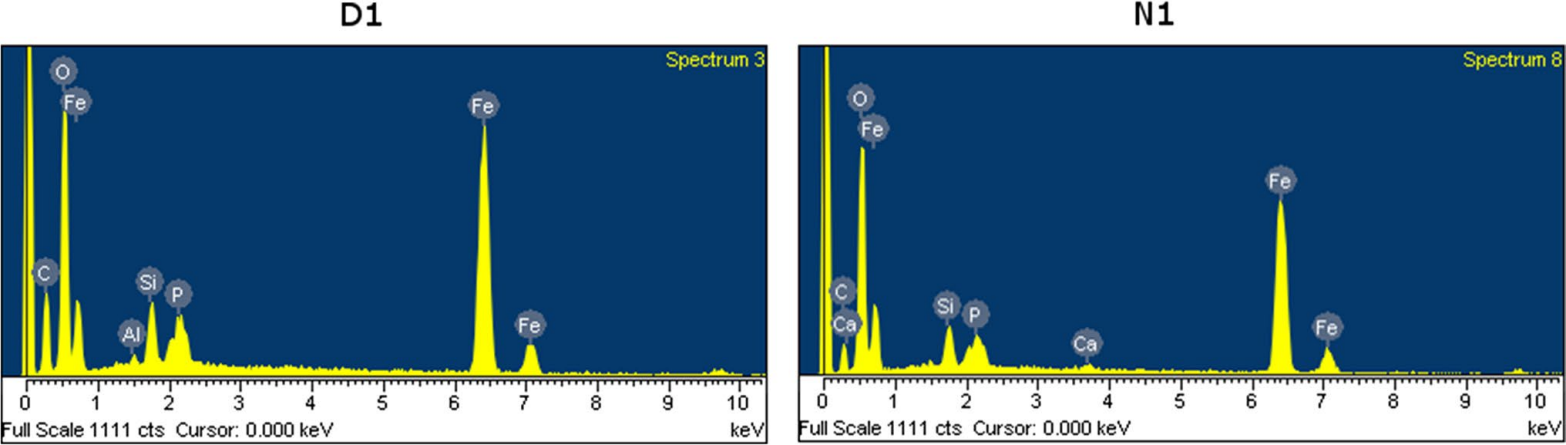
EDX spectra of samples D1 and N1

EDX spectra (Fig. 4) of the two samples of ME-nFe having higher BET surfaces (N1 and D1) show similar elemental compositions with Fe, C, and O as the most abundant elements. The high concentration of O could suggest the presence of iron oxides but also other functional groups that are bonded to the carbon, forming the shell of the nanoparticles. Of course, other characterization techniques would be able to provide a better knowledge of the crystalline structure and the phase of the elements forming the nanoparticles. The carbon content is less pronounced in sample D1 than N1 (28 and 11%, respectively). Since N1 was produced at a higher temperature (and pressure), the gaseous by-product (made of CO_2_ and CH_4_ among others) formed during HTC could have been a little more consistent. On the contrary, oxygen and iron are more abundant in sample N1 (40 and 44%, respectively) than D1 (34 and 34%, respectively). The higher temperature of N1 could be responsible for higher iron incorporation in the solid product. Of course, analysis of the hydrothermal carbonization liquid fraction could help to better understand the mass balance of the process. Si, P, and Ca were also detected in both samples even if their abundance is less significant (lower than 3%). While P and Ca are common elements of green microalgal biomass, Si probably came from diatoms (brown microalgae characterized by a silica shell) that sometimes can be found in small numbers in the microalgal suspension of the Bresso pilot reactor. Nitrogen was not found among the other elements, which is interesting, suggesting that the N content (originally present in the biomass and in the iron nitrate) should be found in the liquid fraction of HTC.

**Fig. 5:**
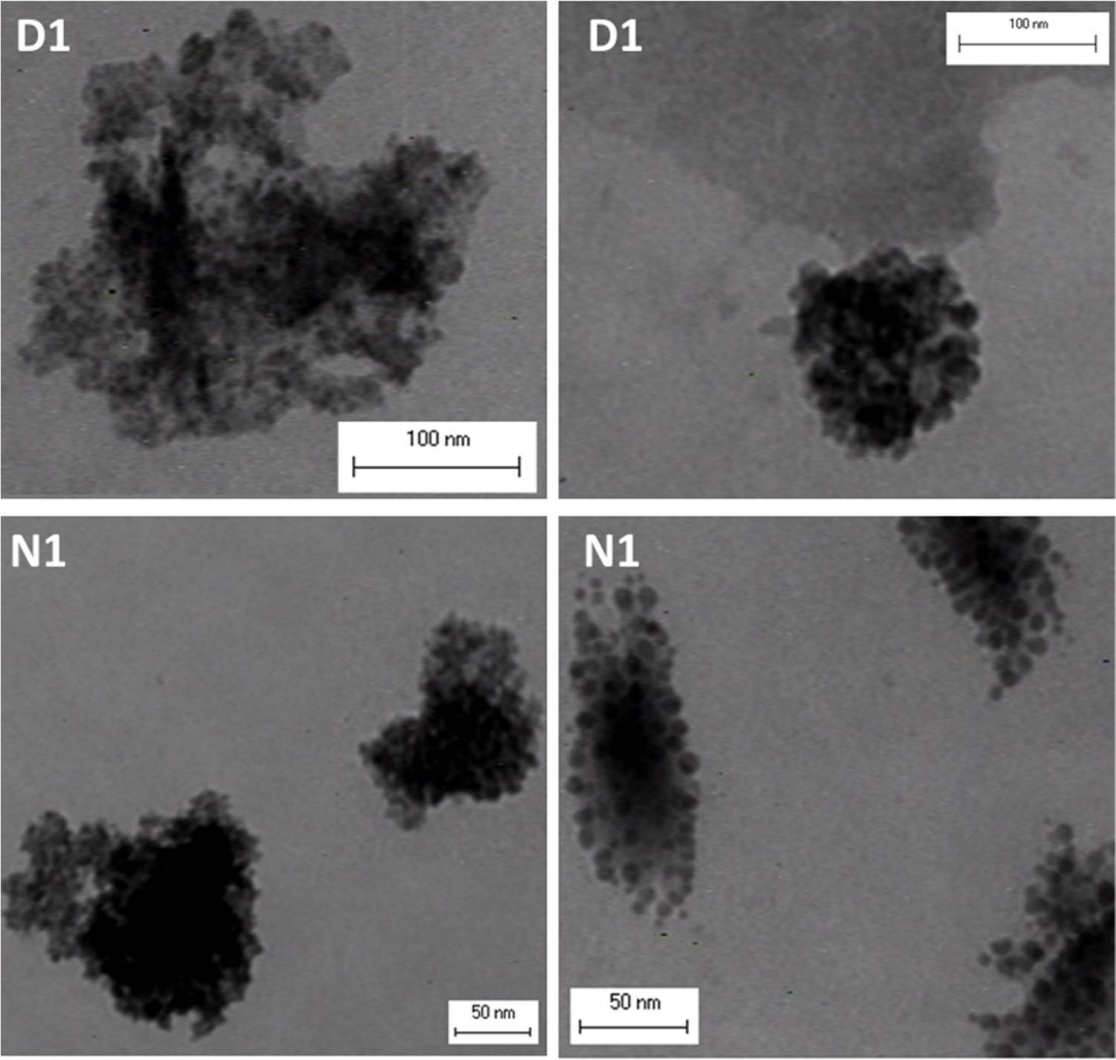
TEM images of samples produced with iron nitrate Fe/C=0.2 at 180 °C (D1, upper squares), and at 225 °C (N1, lower squares)

The samples were also observed at the TEM microscope to detect differences in the structure of the nanoparticles and the iron distribution within the mass of the ME-nFe. The comparison between samples D1 and N1 is shown in Fig. 5, where the more electron-dense areas of the photos (representing the iron as other components detected by the EDX are less meaningful) seem well distributed into the overall masses. Furthermore, also the iron content seems higher in sample N1, confirming the founding of the gravimetric analysis and EDX.

### Selection of the best ME-nFe

By combining the magnetic properties (tested with a neodymium magnet) with the Fe^0^, Fe _tot_, BET surface area, and morphology, the best samples were selected. These were D1 and N1, produced both with iron (III) nitrate nonahydrate with Fe/C molar ratio of 0.2, differing only for the process temperature which was 180 °C for D1 and 225 °C for N1. The properties of the two samples are summarized in Table 5.

**Table 5:** Properties of samples D1 and N1

Sample	[Fe/C]	*T* (°C)	Salt	BET (m^2^g^−1^)	Pore volume (cm^3^g^−1^)	% Fe^0^	% Fe _tot_	% Fe^0^/Fe _tot_
D1	0.2	180	Fe (NO_3_)_3_ · 9H_2_O	120	0.65	8.7	42.7	20.4
N1	0.2	225	Fe (NO_3_)_3_ · 9H_2_O	110	0.58	8.3	66.6	12.5

### Application of the ME-nFe for heavy metal removal

**Fig. 6:**
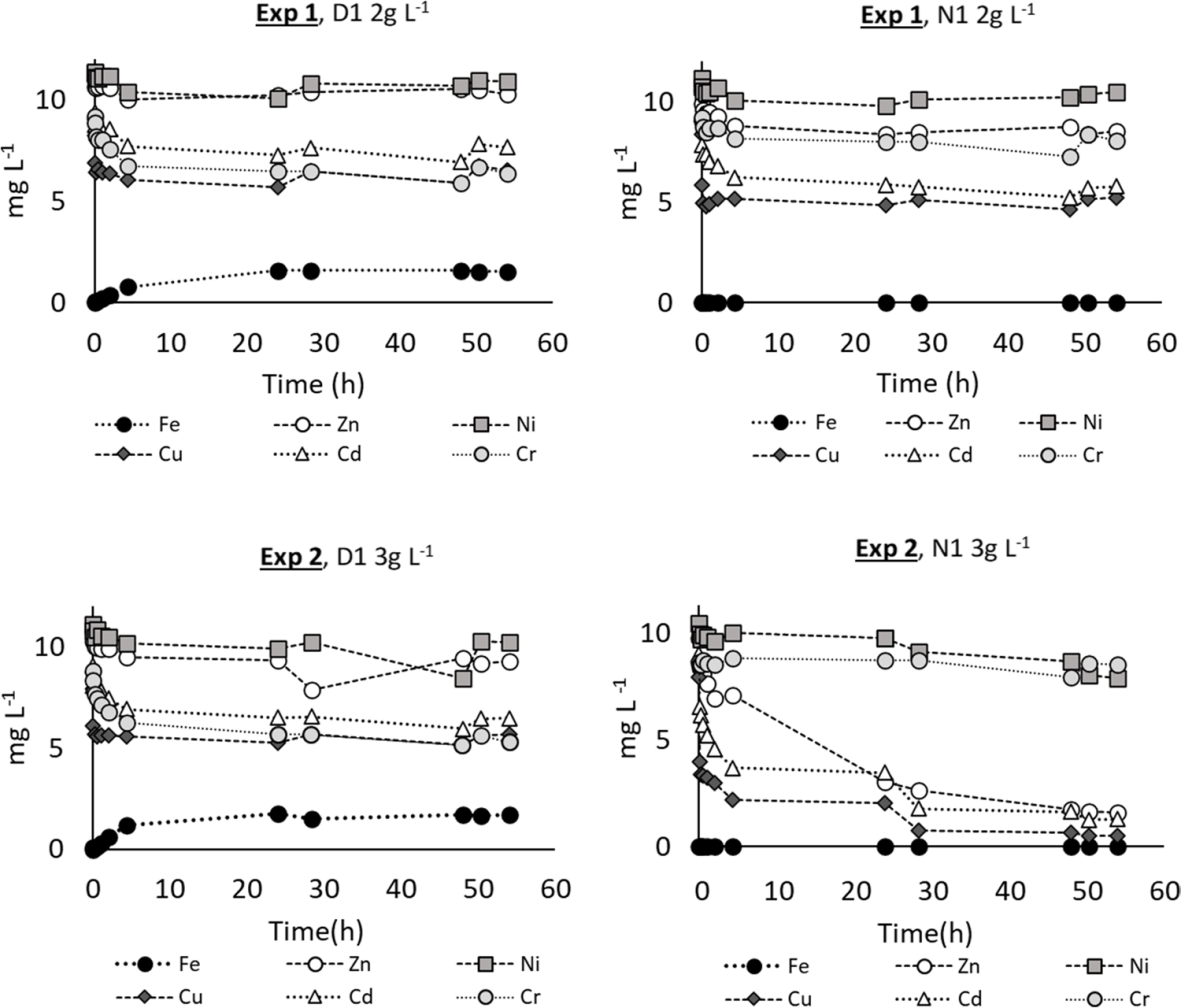
The trend of the residual metal concentrations during the adsorption tests. In the first row, Exp. 1 compares sample D1 and N1 at the sorbent dose (C_n_) of 2g L^−1^. In Exp. 2 the sorbent dose (C_n_) was increased up to 3g L^−1^

**Fig. 7:**
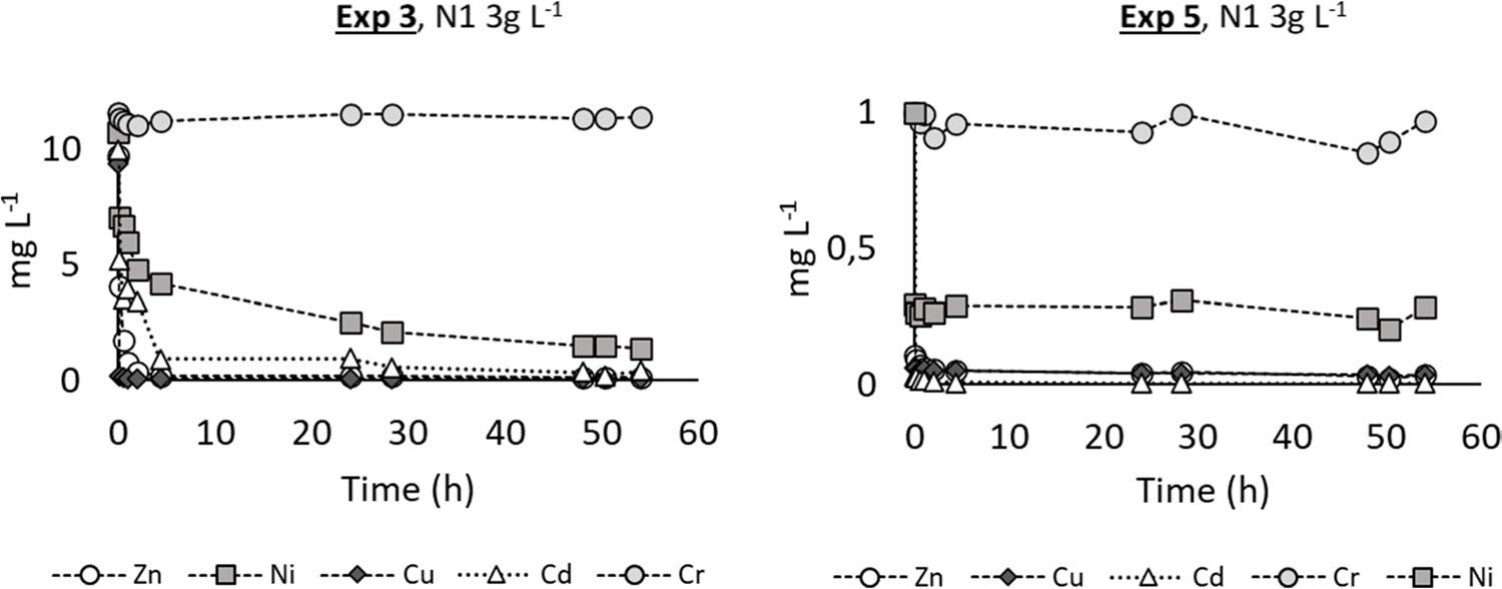
Trend of the residual metal concentrations during the adsorption tests. Exp. 3 and Exp. 5 were done only with sample N1 at different starting metal concentrations with C_n_=3g L^−1^

**Fig. 8:**
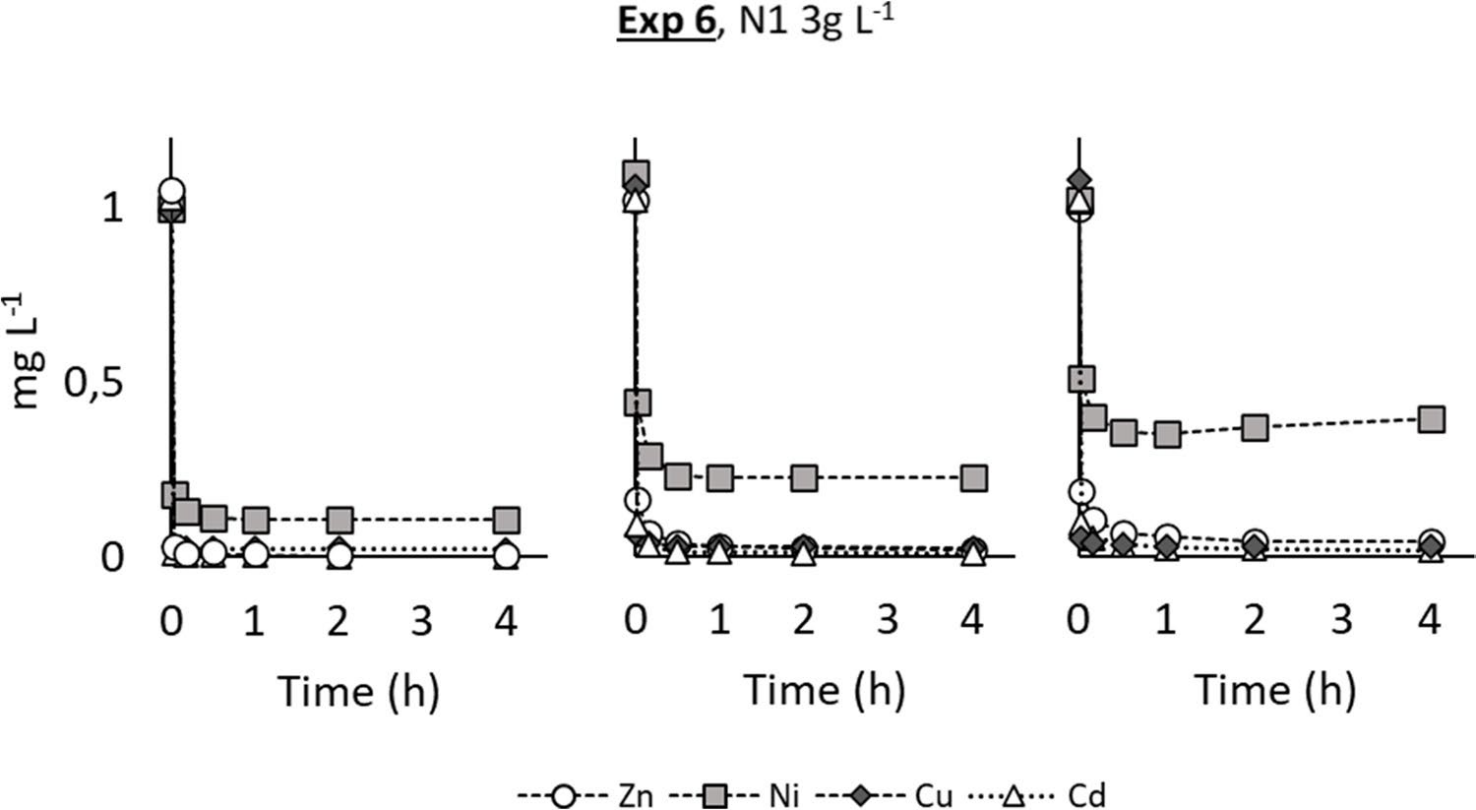
Trend of the residual metal concentrations during the consecutive adsorption tests using the same sample N1, recovered after every single Jar test, with a C_n_=3g L^−1^

The selected samples (D1 and N1) were tested to remove cadmium, copper, zinc, chromium, and nickel from aqueous solutions and, then, from the treated effluent effluents. The test conditions are reported in Table 6 as well as the average results of the three replicates. C_i0_ is the starting concentration of the heavy metals in the solution, C_n_ is the concentration of nanoparticles used, pH_0_ is the pH value of the solution at the beginning of the test. The trends of metal concentrations in the different tests are represented in Figs. 6, 7, and 8.

**Table 6:** Summary of the adsorption test conditions and results. All the tests were done in triplicate except for exp 6, where every single phase was done in duplicate

Test	Sample	Conditions	Removal efficiency (%)				
			Zn	Cu	Cd	Ni	Cr
Exp. 1	D1	C_i0_ = 10 mg L^−1^, pH_0_ = 5.3 C_n_= 2 g L^−1^,	6.2	20.8	18.3	3.6	30.3
		54-h test; Stirring= 90 rpm	±3.5	±4.6	±1.4	±1.7	±13.2
	N1	Water solution	19.2	37.3	38.2	5.9	11.4
			±3.2	±19.5	±2.8	±1.7	±7.6
Exp. 2	D1	C_i0_ = 10 mg L^−1^, pH_0_ = 5.2 C_n_= 3 g L^−1^,	13.7	28.2	28.4	8.3	39.9
			±2.5	±2.0	±4.1	±1.1	±6.6
	N1	54-h test; Stirring= 90 rpm	83.4	93.7	85.8	24.5	1.4
		Water solution	±1.2	±0.4	±1.0	±5.5	±0.4
Exp. 3	N1	C_i0_ = 10 mg L^−1^, pH_0_ = 7 C_n_= 3 g L^−1^,	98.5	99.6	97.2	85.2	2.6
		54-h test; Stirring= 90 rpm	±0.1	±0.2	±0.8	±1.2	±0.9
		Bresso effluent					
Exp. 4	N1	C_i0_ = 10 mg L^−1^ of Cr, pH_0_ = 5.4 C_n_= 3 g L^−1^,	/	/	/	/	19.4
		54-h test; Stirring= 90 rpm					±4.23
		Water solution					
Exp. 5	N1	C_i0_ = 1 mg L^−1^ , pH_0_ = 7 C_n_= 3 g L^−1^,	97.8	96.4	99.6	80.3	12.4
		54-h test; Stirring= 90 rpm	± 0.8	±0.6	±0.1	±5.4	±11.0
		Bresso effluent					
Exp. 6	N1	C_i0_ = 1 mg L^−1^ , pH_0_ = 5.4 C_n_= 3 g L^−1^,	99.4	97.8	99.8	89.4	/
		4-h test; Stirring= 90 rpm	98.7	97.7	99.3	79.2	/
		Water solution	96.2	97.4	98.4	61.3	/

Experiments 1 and 2 were made to compare the overall performance of the two samples, having as the sole difference the production process temperature. The ME-nFe produced at 180 °C (sample D1) resulted to be inadequate for the use it was designed for, as it released a relevant amount of iron: at the end of the experiments an iron concentration of 1.5 mg L^−1^ was reached, suggesting that the iron was not fully encapsulated in the carbon matrix. The ME-nFe produced at 225 °C (sample N1) did not present the same problem. This confirms that temperature is a crucial factor to produce iron nanoparticles through the HTC process (Nizamuddin et al. 2017; Lu et al. 2013). The best achievement with the lowest ME-nFe dose (2 g L^−1^) was a 40% removal for copper and cadmium. However, no release of metals to the solution occurred, even after a very long time. Concerning the standard deviations of some efficiency data, the variability could be explained by the possible presence of aggregates in the sample of ME-nFe used for the different Jar tests. It can also be noted that low removal efficiencies are characterized by higher standard deviations. Better results were obtained with sample N1 at 3 g L^−1^, as shown in Fig. 6 (Exp. 2), while no improvement was observed for D1, still releasing iron. The use of 3 g L^−1^ of N1 allowed the removal of 93%, 86%, and 83% for copper, cadmium, and zinc, but the effect was negligible for nickel and chromium. The increase of the ME-nFe dose was proved to be an effective strategy to improve the overall removal of heavy metals. Exp. 2 was also useful to clarify that the effectiveness of the nanoparticles does not depend only on the surface area, which was higher in sample D1 but on the combination of different characteristics such as the total iron incorporation and its distribution within the solid product. The differences between the two nanoparticles highlighted by TEM analysis and iron determination might explain the better removal performances of sample N1 that was thus chosen for the subsequent experiments.

Exp. 3 (Fig. 7) was carried out in the same conditions as Exp. 2 but using the treated effluent from Bresso WWTP instead of Milli-Q® water to prepare the starting metal solutions to test the behavior of the nanoparticles in a realistic situation and to observe if and how the nanoparticles interact with the other dissolved and suspended components of the effluents. In that case, the results were the best for all the added metals, except for chromium, with removal efficiencies of 99.6%, 98.5%, 97.2%, and 85.2% for copper, zinc, cadmium, and nickel, respectively. Such better performance could be due to interaction between the heavy metals, the dissolved solids, and the inorganic anions of the wastewater, leading to a better availability for the adsorption on the surface of the nanoparticles. Also, a natural increase of pH was observed during the trial (as for the other experiments where starting pH was around 5), from the starting value of 7 to the final value of 8.4 which might have favored hydroxide precipitation and adsorption in the core of the nanoparticles. The increase in pH can be explained by the redox reactions involving zero-valent iron in a water system. Fe^0^ is oxidized to Fe^2+^, H^+^ is consumed, while OH^−^ is released. On the other hand, chromium removal was still minor. According to Zhuang et al. (2014), the surface charge of the nanoparticles changes according to the pH of the solution to be treated. The point of zero charge of the Me-nFe (PZC), the pH at which the surface of the nanoparticles has a neutral charge, was 6.4. When pH is higher, the overall charge of the ME-nFe becomes negative. Calderon and Fullana (2015) reported that in alkaline solution an overall negative charge on the nanoparticle’s surface can determine a low removal and reactivity for dichromate, and this could explain the results obtained in our experiments. So, a Jar test (Exp. 4) was conducted at a lower pH (5.4) on a water solution containing only chromium, to avoid possible interferences among heavy metals, but the outcome (19.4% average removal efficiency), even if better than the previously obtained one (1.4%), was still not satisfying. It should also be underlined that K_2_Cr_2_O_7_ dissolves in water forming dichromate anions that have different chemisorption compared with the other cation metals that were so easily adsorbed. Exp. 5 (Fig. 7) was characterized by a lower contaminant concentration (1 mg L^−1^ for each heavy metal cation) to represent a more realistic scenario. Even with this configuration, the results are comparable with the one of Exp. 3 with an overall removal of 99.6%, 97.8%, 96.4%, 80.3%, and 12.4% for cadmium, zinc, copper, nickel, and chromium respectively. The outcome suggests that the ME-nFe were not affected by the starting concentration of contaminants, at least in the tested range. The difference in the starting pH in experiments 3 and 5 with respect to the others is due to the use of Bresso effluents as the matrix to prepare the starting solution to be tested. No pH adjustments were performed.

The last Jar test (Exp. 6), whose results are shown in Fig. 8, had the main goal to understand if the same nanoparticles could be re-used for subsequent treatments without losing their effectiveness due to the saturation effect. Considering the negligible removal in the previous experiment, chromium was excluded from the starting solution. The same nanoparticles were indeed used (after recovery and dewatering) for three consecutive Jar tests. Every single phase lasted only 4 h (that was the time when the equilibrium adsorption was reached in the previous experiments) since the release of the cations back to the treated solution was excluded. Even if the performance slightly decreased after every usage, the removal efficiencies were still over 96% for zinc, copper, and cadmium, while nickel removal decreased from 89 to 79% in the second test and 61% in the third one. The lower removal of nickel was not unexpected as nickel had always shown a smaller affinity for the nanoparticles, so it is likely that it was less competitive than the other tested metals when the availability of active sites decreased.

As suggested by literature concerning similar applications (Calderon et al. 2018; Kharisov et al. 2012), the mechanism leading to the heavy metals remediation through the use of the carbon-encapsulated iron nanoparticles should involve at least two steps: the carbon matrix (deriving in this study from the microalgal biomass) which supports the iron nanoparticles provides a high adsorption capacity on the external layer, favoring metal sequestration, especially for the ones having a reduction potential (E^0^) more negative than the one of Fe (like Zn^2+^ and Cd^2+^); the second mechanism could lead to the reduction of the heavy metals having higher E^0^ than Fe (Cu^2+^and Ni ^2+^) by the zero-valent iron or the complexation with the iron oxide or other functional groups on the surface of the nanoparticles. This mechanism could also happen at the same time (Kharisov et al. 2012). Of course, a better understanding could be achieved by characterization techniques such as XRD and FTIR to compare the characteristics of the nanoparticles before and after their use. However, understanding the mechanism of removal of heavy metals is beyond the scope of this paper, as the goal was to first understand if the microalgae were a feasible precursor to produce iron-loaded adsorbents through HTC for wastewater treatment purposes.

The result obtained in the Jar tests were promising even if the ME-nFe should also be tested on lower concentrations of contaminants (μg L^*−*1^) in the future. Comparing the obtained performance with literature is not easy as microalgae were never used to produce iron nanoparticles to remove heavy metals before and experimental adsorbents are often obtained with different chemical processes (such as pyrolysis). The protocol to produce the ME-nFe was indeed similar to the one described in Calderon et al. (2018). Of course, their starting feedstock was different, consisting of olive mill wastewater. Is important to highlight that the final properties of the nanoparticles are strongly influenced by the used biomass. The mentioned authors used their nanoparticles, produced at 200 °C, for the removal of zinc, nickel, copper, cadmium, and chromium, testing their effectiveness with and without post-treatment (consisting in the activation of the nanoparticles under N_2_ to increase the zero-valent iron content). The first type of nanoparticles was used, testing a load of 1 g L^*−*1^ to treat a solution containing 10 mg L^*−*1^ of each heavy metal. The removal efficiency was 34.1, 39.9, 30.4, 87.7, 88.5% for Zn, Ni, Cd, Cu, and Cr, respectively. Those results are better than the one obtained in this paper when the lower dose was used (Exp. 1) but worse than the one in experiment 2 (apart from Cr and Cu). The authors also used the nanoparticles after the pretreatment to increase the zero-valent iron content, testing a sorbent concentration of 2.1 g L^*−*1^ which gave far better results (removal efficiency >98% for all the heavy metal cations). However, the second test should have been performed with the same sorbent concentration as the previous one, allowing us to understand if the improvements were due to the increased load of sorbent or to the post-treatment of the nanoparticles.

## Conclusion

The possibility to valorize the microalgal biomass grown on the centrate from municipal sewage sludge as a starting material to produce iron nanoparticles useful for polishing the final effluent was confirmed at the laboratory scale. The microalgae were adequate for the hydrothermal carbonization process, which is considered an environmentally friendly way to produce iron nanoparticles. A protocol for a single-step production was applied to combine the carbonaceous structure of the biomass to an iron salt so that the forming iron nanoparticle could be incorporated into the carbon matrix and be protected from too fast oxidation. The goal was to produce a nano-porous material with a high sorption capacity (due to the carbon shell) and reducing properties (due to the iron oxide and zero-valent iron nanoparticles). Different samples were prepared, changing the process conditions, using two iron salts, three different temperatures, and four ratios between iron and biomass. Considering the characterization in terms of magnetism, BET surface area, total iron, and zero-valent iron content, the two best prototypes were selected which mainly differed for the temperature of synthesis. After full characterization of the properties of the nanoparticles, two samples were tested as adsorbents for metal removal, and only one of them showed adequate performance: this demonstrated the great importance of the operation parameters of the HTC process. Using 3 g L^*−*1^ dose, the removal of copper, zinc, cadmium, and nickel in Jar tests was fully satisfying at high (10 mg L^*−*1^) and low (1 mg L^*−*1^) starting concentrations, both in water and in the treated effluent from Bresso WWTP, and no iron release was observed during the trials. The removal was always a little less efficient for nickel than for the other metals, but, unexpectedly, chromium was removed to a negligible extent. The reason for such different performances on chromium will need further studies to be understood. In view of scaling up, an important preliminary achievement was the effectiveness of recovered and recycled nanoparticles for 2–3 adsorption trials. The only metal whose removal decreased significantly with time was nickel, and the reasons for that will also need further investigation. The possibility of easy magnetic separation and re-use of the nanoparticles is very important in terms of energy and money-saving and for the environmental impact of the production and final disposal. The study led to interesting new scenarios concerning the use of microalgae for wastewater treatment strategies. Microalgae-bacteria consortia could be integrated into real WWTP as an alternative biological treatment to remove contaminants from wastewater, and the obtained biomass could be exploited to synthesize ME-nFe to be used directly in the plant for a tertiary polishing treatment of the effluent.

## Data Availability

The data that support the findings of this study are available from the corresponding author, M.M., upon reasonable request.
